# River Tea Tree Oil: Composition, Antimicrobial and Antioxidant Activities, and Potential Applications in Agriculture

**DOI:** 10.3390/plants10102105

**Published:** 2021-10-04

**Authors:** Mursleen Yasin, Adnan Younis, Talha Javed, Ahsan Akram, Muhammad Ahsan, Rubab Shabbir, Muhammad Moaaz Ali, Ayesha Tahir, Enas M. El-Ballat, Mohamed S. Sheteiwy, Reda Helmy Sammour, Christophe Hano, Fahad A. Alhumaydhi, Mohamed A. El-Esawi

**Affiliations:** 1 Institute of Horticulture Science, University of Agriculture, Faisalabad 38000, Pakistan; mursleenyasin@gmail.com (M.Y.); adnanyounis@uaf.edu.pk (A.Y.); ahsanakram66@gmail.com (A.A.); muhammadmoaazali@yahoo.com (M.M.A.); 2 College of Agriculture, Fujian Agriculture and Forestry University, Fuzhou 350002, China; mtahaj@fafu.edu.cn (T.J.); rubabagronmy123@yahoo.com (R.S.); 3 Department of Agronomy, University of Agriculture, Faisalabad 38000, Pakistan; 4 Department of Horticulture, The Islamia University of Bahawalpur, Bahawalpur 63100, Pakistan; ahsan.horti@iub.edu.pk; 5 Seed Science and Technology, University of Agriculture, Faisalabad 38000, Pakistan; ayeshatahir643@gmail.com; 6 College of Horticulture, Fujian Agriculture and Forestry University, Fuzhou 350002, China; 7 Botany Department, Faculty of Science, Tanta University, Tanta 31527, Egypt; enas.elballat@science.tanta.edu.eg; 8 Department of Agronomy, Faculty of Agriculture, Mansoura University, Mansoura 35516, Egypt; salahco_2010@mans.edu.eg; 9 Department of Botany and Microbiology, College of Sciences, King Saud University, P.O. Box 22452, Riyadh 11495, Saudi Arabia; rsammour@ksu.edu.sa; 10 Laboratoire de Biologie des Ligneux et des Grandes Cultures (LBLGC), INRAE USC1328, Université d’Orléans, 28000 Chartres, France; hano@univ-orleans.fr; 11 Department of Medical Laboratories, College of Applied Medical Sciences, Qassim University, Buraydah 51452, Saudi Arabia; f.alhumaydhi@qu.edu.sa

**Keywords:** essential oils, tea tree oil, antioxidant, antimicrobial

## Abstract

Melaleuca is one of the genera of the Myrtaceae family enriched in tea tree oil (TTO). Tea tree oils of *Melaleuca bracteata* and *Melaleuca alternifolia* are of prime importance and have antioxidant and antimicrobial properties. Terpinen-4-ol and 1-8 cineole are major constituents of *M. alternifolia* oil. The percentages of the compounds in the oils can slightly vary according to the region of plant harvest, the distillation technique, or the part of the plant used for oil extraction. TTO has a bactericidal effect against various bacterial species such as *Bacillus cereus*, *B. subtilis*, *E. coli*, *Pseudomonas putida*, and *S. aureus*. Several reports proved that this essential oil is also effective against fungal strains of *Fusarium*, *Aspergillus*, and *Candida* species. It also has antioxidant properties such as radical scavenging activity and reducing power. The antioxidant properties of TTO at a concentration of 30 mM were observed to be greater than those of butylated hydroxytoluene (BHT), commonly used as a synthetic antioxidant. TTO is also an effective organic fungicide, herbicide, and insecticide for use in the agriculture sector. Postharvest application of the oil has been found efficient on sweet basil, citrus, and strawberry. It is concluded that tea tree oil has the potential to be used in the food, agriculture, and pharmaceutical industries as a natural antimicrobial and preservative agent. This review provides comprehensive information regarding the antioxidant and antimicrobial activities of tea tree oil and its potential applications in agriculture.

## 1. Introduction

The natural products of plants such as essential oils (EOs) are highly valued worldwide. EOs are complex volatile compounds extracted from roots, branches, twigs, leaves, flowers, fruits, buds, and seeds, which are stored in glandular trichomes, cavities, canals, epidermal, and secretory cells [1,2]. The applications of EOs are continuously increasing due to their insecticidal, antifungal, antiviral, antibacterial [1], anti-inflammatory [2], anti-microbial [3], anxiolytic, and antidepressant properties, tested in vitro [4]. Various chemical compounds such as flavonoids (quercetin), glycosides (chrysoeriol 7-*O*-glucopyranoside), alkaloids (terpinene), and phenolics (thymol) are responsible for the above-mentioned properties of EOs [2,5]. 

The Myrtaceae family is one of the main essential oils-enriched plant families, and *Melaleuca* is a well-known genus of this family with a high content of EOs [2]. *Melaleuca* is an indigenous genus of Australia that can also adapt to various agro-climatic zones. Major species of this genus such as *M. alternifolia, M. quinquenervia, M. bracteata*, and *M. cajuputi*, are subjected to oil extraction, and their essential oil is known as tea tree oil (TTO) worldwide [2,6]. In World War II, TTO was utilized as an insect repellent and a general anti-microbial agent [7]. Later, its applications have expanded, and its popularity in various industries is currently soaring as it is used such as a preservative, fungicide, natural biocide and in cosmetics, aromatherapy, allopathic and herbal medicines, etc. [2]. TTO is transparent to slightly yellow in color, with a cooling effect similar to that of menthol and a strong odor just like camphor [8,9]. TTO is composed of approximately 100 various chemical substances, predominantly monoterpenes (terpinolene, α-pinene, 1,8-cineole, p-cymene, γ-terpinene, and terpinen-4-ol) and sesquiterpenes, with their respective alcohols (alcohol terpinol, monoterpenes, etc.) [2,10]. TTO extracted from *M. bracteata* also contains phenylpropanoids and methyl eugenol as major components [2]. Terpinolene (17.5%), p-cymene (12.8%), and γ-Terpinene (15.2%) are the major components of *Melaleuca buseana* leaf oil, whereas *Melaleuca dawsonii* leaf oil has α-phellandrene (10.3%) and α-pinene (12.8%) as principal components. In *Melaleuca gnidioides,* spathulenol (14.7%), β-pinene (13.6%) and α-pinene (23.6%) are the major components. *Melaleuca brevisepala* and *Melaleuca sphaerodendra* var. *microphylla* produce oils in which β-caryophyllene (26.8% and 28.8%, respectively) is the principal component. β-caryophyllene (14.1%) and α-pinene (24.8%) are the principal compounds recorded in *Melaleuca pancheri* leaf oil. *Melaleuca brongniartii* oil contains limonene (19.8%), β-pinene (10.3%), and α-pinene (23.3%) as the principal monoterpenes [11].

The properties of *Melaleuca* have not been investigated in detail in Pakistan, although this plant is cultivated across Punjab, particularly *M. bracteata* [2]. Similar to other EOs, TTO also has medicinal, insecticidal, antifungal, and cytotoxic properties [12,13]. The literature indicates that TTO can be used in the agriculture sector as an organic pesticide, bactericide, fungicide, or weedicide to decrease pathogen and weed attack on crops, prevent food spoilage, enhance the vase life of flowers, and improve the shelf life of fruits and vegetables [2]. This review provides comprehensive information regarding the antimicrobial and antibacterial activities of tea tree oil and its potential applications in agriculture.

## 2. Essential Oils

The history of mankind indicates that human survival is dependent on seeds and plants. Essential oils have played an important role in history since 5000 BC in India and since 3000 BC in Mesopotamia and Greece [14,15]. In the last decade, the market demand of essential oils has been increasing because of their elevated uses in perfumes, cosmetics, aromatherapy, foodstuff, pharmaceuticals, and also in agriculture [16,17]. To meet the higher consumer needs, almost 40 to 60 thousand tons of essential oils are being produced and processed annually, making a total worth of 700 million dollars [18]. Their price depends on plant quality, extraction method, and area of application.

Essential oils (EOs) are naturally significant for plants themselves as a part of their communication and defense mechanisms. Being a plant’s natural defense, they protect plants from microbes and herbivores. EOs can play a role in pollen and seed dispersal by attracting beneficial insects and repelling harmful ones [19]. EOs can also regulate plant thermo-tolerance [20]. The fragrance or smell and flavor of aromatic plants is also due to the presence of these secondary metabolites [21] in secretory structures located in different plant organs [22].

### 2.1. Uses of Essential Oils

Plant extracts and EOs have been consumed for diverse purposes for thousands of years [23]. Their usefulness is broad, including the fields of agriculture, food, pharmaceuticals, cosmetics, biomedicals, and veterinary products [7]. For instance, the oils from cedar-wood and rosewood re used in perfumery, while those from fennel, lime, and berry are used as flavoring agents for drinks [24]. Moreover, lemongrass oil is used as a postharvest preservative for food crops [25]. These oils are also used in folk medicine and dietary regimens [26] and have been suggested for disease treatment by several reports [27], in particular as anticancer, antiprotozoal, antiviral, antibacterial [28], antioxidant, and antifungal agents [29].

### 2.2. Compositional Characteristics

The characteristics of essential oils depend on their composition, which in turn may be altered by extraction techniques and be influenced by the geographical area of plant harvest, season [20], and plant genetics [30] and developmental stage [31]. Moreover, the chemical nature of aromatic oils can vary in different parts of the same plant. Researchers found that the EO extracted from the seeds of coriander has a different composition with respect to the oil obtained from immature leaves [32]. The antibacterial properties of volatile oils are mainly due to phenolic compounds [33]. Studies revealed that the main antibiotic components in thyme are thymol (10–64%), carvacrol (2–11%), and p-cymene (10–56%) [34]. The antibacterial trait is due to eugenol (75–85%) in clove [35], carvacrol and g-terpinene in oregano [36], linalool in coriander [32], and a-pinene in rosemary [37]. Antibiotics also formed from the precursors p-cymene, g-terpinene, and thymol in essential oils from *Origanum* and *Thymus* species [38,39]. The varying composition of EOs also affects their germicidal properties. It has been found that the volatile oil extracted from herbs forthwith after flowering has more robust antimicrobial properties [36]. The enantiomers of thymol, terpinene, eugenol, linalool, etc., in EOs also contribute to their antimicrobial effects up to certain levels [7]. The extraction method affects this trait, as it observed seen that essential oils obtained by hexane extraction have a higher antimicrobial potential than those obtained by steam distillation [40].

## 3. Tea Tree Oil (TTO)

The *Melaleuca* genus of the Myrtaceae family is composed of 230 species that are widely distributed across the globe, largely in Australia, tropical America, South Asia, and Indonesia [41]. It includes many species enriched in essential oil, i.e., *M. alternifolia*, *M. bracteata*, *M. quinquenervia*, *M. leucadendra* L., *M. viridiflora*, *M. acacioides*, *F. Muell*., *M. alsophila*, and *M. argentea* [42]. However, the most remarkable species for the production of commercial tea tree oil are *M. cajuputi, M. alternifolia*, *M. quinquenervia*, and *M. bracteata* [6,11,43]. The essential oil obtained from the genus *Melaleuca* is commercialized under the label of “Tea Tree oil” (TTO). This oil is also known by other common names such as ‘Cajupat oil’ or ‘Australian tea tree oil’ because the plant species producing it are native to that region. The oil is transparent to slightly yellow in color, with a cooling effect similar to that of menthol and a strong odor just like camphor [9]. The relative density of this oil is 0.885–0.906, it can be dissolved in organic solvents like alcohol and acetone but is lowly soluble in water. These plants can adapt in various ecosystems and diverse climates [44]. Reports in the literature indicate that these species are highly stress-tolerant because they accumulate an organic compound called proline betaine [45]. 

Plant parts and their essential oils have been utilized as folk medicines by Aboriginals [46]. In New South Wales, the leaves of the tea tree have been used in traditional medicine since aboriginal times [47]. After the Penfold’s report about the importance of tea tree oil, its utilization greatly increased in the second and third decades of the 20th century [48]. Since 1920, in Australia, it has been used as an antiseptic [48]. This natural oil is an efficacious bactericide, fungicide, and antiseptic, it is quite safe and effective, and has potential applications in the health and cosmetic industries [49].

### 3.1. Production of TTO

The production and distillation of TTO originated in Australia. The industry started to produce TTO commercially after the report of Penfold in the 1920s, which presented a survey of Australian essential oils [47,50,51]. At first, it was obtained from *M. alternifolia* by distillation, which was often done on the spot in wood-fired bush stills. This method continued for some decades but was abandoned after the discovery of antibiotics. The industry revived its commercial importance in the 1970s, and plantations were established on a large scale to support the production of TTO for commercial use [52,53]. The extraction protocol is hydrodistillation according to the British Pharmacopeia 1963 [54]. 

Australia is the biggest producer of TTO and ranks first in its research and development. This industry has now progressed to such a maturity level that an association for TTO production has been created, named Australian Tea Tree Industry Association Ltd. (ATTIA). The annual production of TTO in Australia is 900 tones, worth 35.32 million dollars [55]. Ninety percent of the oil from Australia is exported to the USA (54%), Europe (30%), and Asia (14%). In addition, 80% of the oil is exported in bulk form, and a small fraction is used in value addition [55]. China is the second largest producer of this oil and a strong competitor of the Australian market because of its economical production technologies. However, Australia meets the international standards by its high quality and uniform production. Other countries producing TTO are South Africa, Kenya, Indonesia, and Thailand, which mainly produce it from *M. alternifolia* [55].

*Melaleuca* species are native to the coastal regions of Australia, and previously, oil extraction was done from natural stands. However, there were chemical and genetic disparities due to natural variations. To overcome this problem, plantations of *Melaleuca* species were established for the commercial harvesting of tea tree oil. According to the Rural Industries Research and Development Corporation (RIRDC) fact sheet of 2017, there are 80 registered growers of tea tree in Australia and it is cultivated over an area of about 40,000 hectares. Value-added products of tea tree oil are cosmetics, pharmaceuticals, and aromatherapy and veterinary products. Because of its bioactivity, this oil is sold directly or after transformation into antiseptic or antibacterial products [56]. These comprise soaps, creams, toothpastes, mouthwashes, lip balms, acne creams and lotions, etc.

### 3.2. Composition of TTO

Almost 100 types of components in TTO have been identified [57]. It was found that 50% of them are hydrocarbons and 50% are oxygenated compounds. The variation characterizes essential oils from different geographical sources. However, the predominant compounds are monoterpenes, sesquiterpenes, and their respective alcohols [10]. *Melaleuca* species present a multitude of aromatic compounds in their volatile oils [58], including monoterpenoids such as terpinen-4-ol, terpinolene, p-cymene, α-terpineol, 1,8-cineole, α-pinene, and α-terpinene. Sesquiterpenoids in these oils are β-caryophyllene, ledol, (E)-nerolidol, viridiflorol, monoterpene, and alcohol terpineol. Phenylpropanoids in TTO are methyl eugenol and methyl isoeugenol [59].

### 3.3. Tea Tree Oil from Melaleuca alternifolia

The most investigated species of *Melaleuca* for ornamental purposes and essential oil production is *Melaleuca alternifolia*. Under natural conditions, it grows up to 8 m [56]; 3-year-old trees produce whitish- or cream-colored flowers as terminal spikes [60]. The oil from this species is of commercial interest and utilized by different industries [48]. The main components of this oil are hydrocarbons of terpenes which include terpinen-4-ol, alpha and gamma terpinene, 1,8-cineloe, and terpinolene [27]. These are chemical compounds with the formula C_5_H_8_, having a volatile and aromatic nature. In the second half of the 20th century, various compounds have been identified in TTO by different analytical methods [48]. Gas chromatography and GC with mass spectrometry revealed about 100 compounds, with terpinen-4-ol being the chief component accounting for 40% of the total composition [52].

Terpinene and α-terpinene were the second and the third most concentrated compounds, representing 23% and 10% of the total content, respectively. The other main compounds in that oil were identified as 1,8-cineole, terpinolene, p-cymene, α-pinene, α-terpinolene, aromadendrene, limonene, and sabinene [52]. The storage conditions (heat and air exposure, light, and moisture) can alter the chemical composition of TTO, as some compounds such as alpha and gamma terpinene may degrade with time. Therefore, the oil must be stored in a cool, dry, and dark place, most likely in an oil veil to prevent chemical changes [52]. Terpinen-4-ol was shown to inhibit inflammation in activated human monocytes [61]. When tea tree oil and terpinen-4-ol were evaluated on human melanoma cells, they blocked their growth and promoted their death [62]. in Table 1, the compounds identified in the oil in various studies are listed.

### 3.4. Tea Tree Oil from Melaleuca bracteata

*Melaleuca bracteata*, also known as black tea tree or river tea tree, was originally distributed from east to north Australia [66]. There are many commercial cultivars for ornamental purposes, such as ‘Revolution Gold’ and ‘Revolution Green’ [67]. This species grows like a shrub and may reach up to 6–7 m in height. Another common name for *M. bracteata* is Golden Melaleuca, which is commonly used in Pakistan. The plant is commonly known for its ornamental uses in South Africa. In a previous study, continuous secretion of gastric acid was investigated. The results revealed that an *M. bracteata* extract significantly decreased the secretion of gastric acid in a dose-dependent fashion. Furthermore, in animals treated with the extract and misoprostol, the mean ulcer scores were significantly lower compared to those of control animals [68].

The essential oil obtained from the leaves and twigs of *M. bracteata* also has antiseptic properties [68]. It is enriched in methyl eugenol and methyl cinnamate. Though the tea tree oil from this species is not commercialized like the product from *M. alternifolia* species, this essential oil also has antioxidant and antibacterial properties, mainly due to eugenol methyl ether [69]. This essential oil was suggested as a source of natural antibacterial and antioxidant agents after evaluating its effects against food-borne pathogens [70]. The examination of the tea tree oil of *M. bracteata* from various geographic regions indicated that phenyl propanoids are its chief chemical contents. The oil from Egypt was reported to contain 97% of methyl eugenol [71], while that of Australia only 50%. Australian oil also contained methyl isoeugenol, iso-elemicin, and elemicin [64]. 

The EO of this species from Malaysia is also enriched in methyl eugenol (76%), and showed strong fruit fly attractive abilities [13]. Methyl eugenol has also insect repellent properties and the ability to attract flower pollinators. Methyl eugenol was described as a floral synomone (attractant) in the coevolution of the orchid species of the genus *Bulbophyllum* along with fruit flies. It has been recognized as the most effective compound for its insect killing effects and the best insect repellent among the major constituents of EOs [72]. The *Bracteata* oil from India was reported to contain phenylpropanoids, with 89% of methyl eugenol and 2–5% of methyl cinnamate [73]. In addition, betulinic acid and oleanolic acids have been identified in this oil. EOs have anti-inflammatory properties [74]. Further, the presence of 3-hydroxyl-lup-20 (30)-ene-28-oic (betulinic acid, BA) and 3-hydroxylolean-12-ene-28-oic (oleanolic acid, OA) and its acetate in *M. bracteata* oil also imparted anti-ulcer affects, verified in albino rats used as model animals [68]. A gas chromatographic spectrum of *M. bracteata* oil extracted by hydrodistillation in Pakistan [63] was published [2], and the identified compounds are presented in Table 2, with other molecules described in previous studies.

## 4. Antimicrobial Importance of TTO

The essential oil of the *Melaleuca* genus has been utilized by Australia mainly because of its antimicrobial, anti-inflammatory, and anti-candidal traits [75,76]. In tea tree oil (mainly from *Melaleuca alternifolia*), the major components terpinen-4-ol and 1,8-cineole are the main antimicrobial agents [27,48]. This oil has been used as a strong antifungal and antimicrobial agent in soaps, creams, toothpastes, etc. It has also been examined for its effects in various superficial infections such as tinea, acne, oral candidiasis, and cold sores [77]. The oil of *M. bracteata* also exhibited antimicrobial features. The key components responsible for these properties are methyl eugenol and methyl cinnamate [69].

The first antimicrobial trial of tea tree oil was implemented against *Salmonella typhi* (previously known as *Bacillus typhosus*) using the Rideal Walker (RW) test. An RW coefficient of 11 was obtained, indicating that it is 11 times better than phenol [46]. Until the 1970s very few reports were published regarding tea tree oil antimicrobial activity. The minimum inhibitory concentrations against *S. aureus* and *Salmonella* were presented and corresponded to 1:16 and 1:32, respectively [78]. Later in the 1970s, TTO was evaluated against Gram-positive and -negative bacteria along with yeasts and a mold. The least effective concentration for suppressing *S. aureus* was 0.25% [79]. The volatile oil from *Melaleuca* species at a 5% concentration had also passed the Therapeutic Goods Act test for antiseptics and disinfectants [80]. Since the last decade, many studies and reports have been published to support the strong and wide-spectrum antimicrobial, antiseptic, and disinfectant potentials of tea tree oil, as shown in Table 3 and Table 4. Some researchers evaluated the efficacy of the components of the volatile oil showing that their application as single agents can also produce the desirable antimicrobial results. In a study [81], eight compounds from the tea tree oil of *M. alternifolia* were tested against *E. coli*, *C. albicans*, and *S. aureus*. The prime component of the oil, i.e., terpinen-4-ol, exerted an antimicrobial effect against all microbes tested, while alpha pinene and linalool were successful against the first two microbes. Moreover, some reports which suggested that terpinene-4-ol is more effective than the whole oil it derives from [38].

Essential oils are being used as an alternative treatment against multidrug-resistance microbial strains. *Melaleuca* oil was evaluated in different in vitro studies to check its efficiency against different human microbes. In a study, TTO from *M. alternifolia* was tested in vitro against *Candida glabrata, Herpes simplex* virus type 1 (HSV-1), methicillin-resistant *Staphylococcus aureus* (MRSA), and *Pseudomonas aeruginosa* grown in planktonic mode or as biofilms [82]. At a 10% *v/v* concentration and 2.5% MIC, it reduced the survival rate of bacteria in biofilms, damaged *C. glabrata* oxidatively, and diminished HSV-1. In another study, nanoparticles of TTO were tested on a dental biofilm in 38 volunteers [83]. Four solutions were applied done on the fifth day after biofilm formation, i.e., a physiological saline solution (0.85% NaCl) (C^+^), chlorhexidine 0.12% (CHX), *M. alternifolia* oil 0.3% (TTO), and a nanoparticle solution of 0.3% *M. alternifolia* oil (NPTTO). The results demonstrated that NPTTO had a similar antimicrobial effect as CHX and could thus be exploited in the future as a bio-alternative to be used in the treatment or prevention of oral biofilms. TTO form *M. cajupati* was tested against *Mycobacterium tuberculosis* (Mtb), which has now developed a strong resistance against drugs. The oil was tested on of the Mtb H37Rv reference strain and on different clinical strains of Mtb and NTM (non-tuberculous mycobacteria). The MICs of tea tree oil against two multidrug-resistant strains (MDR) of high concern, Mtb-1 and Mtb-10, were 0.5% and 2% (*v/v*), respectively. Therefore, *M. cajupati* oil can be used as an alternative therapuetic approach for drug-resistant microbes [84].

**Table 3:** Antimicrobial spectrum of *M. alternifolia* oil.

Microorganism Tested	* MIC (*v/v* %)	** MBC/MFC (%*v/v*)	Standard	References
*Bacillus cereus*	0.3	-	-	[85]
*Bacillus subtilis*	0.3	-	0.02	[85]
*Escherichia coli*	0.08–2	0.25–4	0.32	[86,87]
*Pseudomonas putida*	0.5	-	0.32	[85]
*Staphylococcus aureus*	0.63–1.25	1.01	0.01	[88]
*Lactobacillus spp.*	1–2	2	-	[89]
*Alternaria spp.*	0.016–0.12	0.06–2	-	[90]
*Aspergillus niger*	0.3–0.4	2–8	>2.00	[79,85]
*Fusarium spp*	0.008–0.25	0.25–2	-	[90]
*Candida albicans*	0.5	0.12–	0.125–0.5	[90,91]
*Aspergillus flavus*	0.31–0.7	2–4	3.12	[92]

* MIC, minimum inhibitory concentration; ** MBC, minimum bactericidal concentration; MFC, minimum fungicidal concentration.

### 4.1. Antibacterial Properties

The anti-bacteria properties relate to the depletion of cell membrane constituents and to the inhibition of bacterial respiration. Most studies to evaluate the oil’s antibiotic potential were performed in *Staphylococcus aureus*, showing that it has an antibacterial activity against this species [93]. The MIC values of tea tree oil against *S. aureus*, *S. epidermidis*, *B. subtilis*, *B. cereus*, *Micrococcus luteus*, *Streptococcus*, *E. coli*, *Pseudomonas*, and *Proteus* ranged between 0.2% and 0.5% volume/volume [85]. *M. alternifolia* oil has bactericidal activity against *E. coli*, *Staphylococcus* species, *Lactobacillus*, and *Actinomyces viscosus*, as reported [87,94,95]. 

The essential oil from *M. bracteata* showed antibacterial activity against *S. epidermidis*, *B. subtilis*, *S. aureus*, and *S. typhimurium*, with an inhibitory zone of 5 to 10 mm [96]. Moreover, it is biostatic against *Pseudomonas aeruginosa*, *Serratia marcescens* strains, and *Halobacterium violaceum* [97]. *B. subtilis* subsp. *spizizenii* growth was also inhibited by this species oil (250 µg/mL), and an inhibition zone up to 44 mm was reported [70]. Methyl eugenol and terpinen-4-ol from both species own efficient antifungal, antibacterial, and anti-nematode properties [13].

**Table 4:** Antimicrobial activity of *M. bracteata* oil [98].

Microorganism Tested	Minimum Inhibitory Concentration (ug/mL)	Zone of Inhibition (mm)
*B. spizizenii*	4	44.0 ± 3.5
*S. aureus*	8	12.8 ± 0.8
*E. coli*	4	16.7 ± 0.3
*P. aeruginosa*	8	12.5 ± 0
*S. enterica*	4	17.8 ± 0.8

### 4.2. Antifungal Properties

Tea tree oil modifies the membrane of fungi, which determines its antifungal activity. The most tested fungal strains are *Candida albicans* and *Candida glabrata* [29]. The effective fungicidal capability of tea tree oil against *Candida*, *Aspergillus*, and *Trichophyton* species was from 0.3% to 1.0% [85]. TTO was analyzed in an experiment consisting in treating invasive fungal wound infections (IFIs) using natural medicine. The results indicated that the oil was very effective as it inhibited the growth of filamentous fungi causing IFIs [99]. It was inferred that TTO at 100% decreased *Fusarium oxysporum* log up to −4 relative to the control only within 15 min of exposure. The exposure time increased from 9 to 15 min with the decrease in the percentage of TTO, i.e., from 100% to 1%. A study proposed that the minimum inhibitory concentration of TTO against *oxysporum* is 0.008–0.25% (*v/v*) [90]. It has also been reported that the inhibition zone produced by methyl eugenol from *Melaleuca* species oil against *Fusarium oxysporum* was 24.3 ± 0.3 mm for the pure oil and 46 ± 0.6 mm for methyl eugenol at a 100 µg/mL concentration [93]. A previous study reported the antifungal properties of tea tree oil extracted from *M. alternifolia* against *Aspergillus flavus* [88]. The concentrations needed for the effective control of the fungus varied between 0.3 and 0.7 (% volume by volume). The oil showed its effectiveness against other *Aspergillus* species such as *A. niger* and *A. fumigatus*, with minimum inhibitory concentrations from 0.016 to 0.4 and from 0.06 to >2 (%*v/v*), respectively [78,90]. 

### 4.3. Antiviral Properties

The antiviral properties of TTO were first tested on tobacco mosaic virus [100]. Later in a field trial, *Nicotiana glutinosa* plants were treated with different concentrations of tea tree oil, corresponding to 100, 250, and 500 ppm. All the plants were then experimentally infected with tobacco mosaic virus. A smaller number of lesions per cm^2^ of leaf were observed on the plants treated with TTO as compared to the control plants. When tested against *Herpes simplex* virus [96], the concentration of TTO controlling plaque formation by 50% was found to be 0.0009% and 0.0008% for HSV1 and HSV2, respectively. TTO was also found to be most effective when applied on the free virus, i.e., before infecting cells. TTO has been found to be effective against bacteriophages. A 50% concentration of TTO reduced the plaques of SA and T7 on lawns of *S. aureus* and *E. coli*, respectively [101]. Therefore, it is concluded that both enveloped and non-enveloped viruses are inhibited by this oil [48].

## 5. Antioxidant Properties of TTO

*M. alternifolia* oil has promising antioxidant abilities [64]. DPPH scavenging activity was reported at 12.5 µg/mL expressed as IC_50_ values (the antioxidant concentration at which 50% of the reaction was inhibited) in comparison to the essential oil of *Eucalyptus globulus* (IC_50_ = 14 µg/mL). The reducing power of *M. alternifolia* essential oil, expressed as CE50 was 24 µg/mL and that of *E. globulus* oil was 48 µg/mL [63]. A previous study also reported that α-terpinene, α-terpinolene, and γ-terpinene have higher anti-oxidative activities than terpinen-4-ol [102]. At a concentration of 10 µL/mL, TTO exhibited better antioxidant properties than butylated hydroxytoluene (30 mM BHT), which is a commonly used synthetic antioxidant [102]. TTO has been proposed as an alternative to maintain oxidative stability of food matrices [103]. The oil has also been found to possess stronger free-radical scavenging abilities and to inhibit lipid peroxidation (with an IC_50_ of 135.9 µg/mL)) in comparison to various natural antioxidants such as quercetin, Vitamin E, and Vitamin C [103]. This property is due to the presence of phenols in the essential oil of tea tree [104]. Moreover, it has been observed that monoterpene hydrocarbons, particularly with activated methylene groups such as terpinene-4-ol and alpha and beta terpinene, are stronger antioxidants compared to sesquiterpenes [105].

Evidence regarding the antioxidant potential of *M. bracteata* was presented [2,106]. The conventional extraction method yielded 19.4 ± 0.2 mg of total flavonoids, 88.6 ± 1.3 mg of total phenolic compounds, and a DPPH scavenging activity of 86 ± 0.3%. Moreover, after optimizing the conditions, the antioxidant values were of 21.6 ± 0.3 mg, 98.7 ± 1.2 mg, and 94.7 ± 0.8% for TFC, TPC, and DPPH, respectively. *M. bracteata* scavenging activity varied from 35.3 to 89.2 ± 0.4%. This activity increased when increasing the oil concentration from 20 to 100 µg/mL [70]. Flavonoids activity was calculated using a calibration curve for rutin and expressed as milligrams of rutin equivalent per gram of dry weight (mg RE/g DW) and that of phenols was represented as milligrams of gallic acid equivalent per gram of dry weight (mg GAE/g DW) [70].

## 6. Applications of TTO in Agriculture

Tea tree oil and its components have been applied in the field of agriculture to prevent food spoilage or in the form of pesticides. Several studies were performed on the basis that TTO is an effective organic fungicide, herbicide, and insecticide that can be used in the context of ‘Green Technology’. It has been found that this oil blocked the mycelial growth of 15 post-harvest fungi. The oil was applied directly as well as in vapor phase. The latter application suggested its use as a fumigant for stored crops [107]. Apart from in vitro trials, some successful field experiments of TTO have also been reported. Powdery mildew in greenhouse cucurbits was controlled with a 1% solution of tea tree oil [108]. Tobacco mosaic virus was controlled by a spray of TTO at 10, 50, 100 and 500 ppm before viral attack. Later, fewer lesions were observed on *Nicotiana glutinosa* plants [100]. *Melaleuca alternifolia* showed effective antifungal control against seed-borne fungi viz. *Ascochyta rabiei*, *Colletotrichum lindemuthianum*, *Fusarium graminearum*, *F. culmorum*, *Drechslera avenae*, *Alternaria radicina*, and *A. dauci* [109]. Moreover, the prolines obtained from *Melaleuca* plants are used to enhance the stress tolerance of economic crops via seed treatments or foliar sprays [45].

Weeds are one of the major causes of production losses in agriculture, comparable to the losses caused by insects and other pathogens together [110]. Chemical control by far is the main method to control weeds. However, intensive and indiscriminate use of synthetic herbicides cause potential health risks and environmental contamination [111]. Moreover, their continuous usage could increase the resistance of weeds to specific chemicals [112]. Therefore, substitutes to chemical control are much needed now. Allelo-chemical compounds like plant extracts and essential oils are proposed as good alternatives [113] because they are biodegradable and less toxic to the environment [114]. The herbicidal effect of tea tree oil from *M. bracteata* was assessed by applying it on *Panicum virgatum*, *Digitaria longiflora*, *Stachytarpheta indica*, and *Aster subulatus*. The first two plants are grassy weeds, while the latter two belong to broad-leaf weed group. At the highest dose of 10 μL/mL, the oil fully controlled seed germination of all weed species. Furthermore, a decrease in chlorophyll content with an increased concentration of oil was observed, which showed that TTO hindered photosynthesis and disrupted the leaf membranes of the weed. Therefore, *M. bracteata* oil could be used as an effective herbicide [115].

Postharvest application of tea tree oil was carried out on sweet basil to enhance its shelf life and quality. Oil was applied in vapor phase at concentrations from 0 to 1 mL/L. The results displayed a significant enhancement of the shelf life of sweet basil from 4 to 10 days. The quality of the herb was also maintained high, with a good water content and high levels of essential oil and chlorophyll in the leaves. Along with these, the antioxidant quality was also elevated, as the peroxidase enzyme quantity increased in the herb after TTO application as compared to the control. The most effective concentration of the essential oil suggested to be used for basil shelf life improvement was 1 mL/L [116]. This volatile oil has also been found to lengthen the shelf life and storage quality of citrus and strawberry when applied in vapor phase [117,118].

## 7. Conclusions

Tea tree oil has gained much fame and become an integral part of the pharmaceutical, agriculture, food, and cosmetic industries in the world. Its importance lies in its complex composition which is characterized by high levels of antimicrobial and antioxidant compounds such as terpinen-4-ol, methyl eugenol, and 1,8-cineole. Hence, it is concluded that tea tree oil has significant potential as a natural microbicide in food and the agriculture industry.

## Figures and Tables

**Table 1:** Compounds extracted from *M. alternifolia*, as reported in different studies.

Identified Compounds	Quantity (%) [63]	Quantity (%) Miscellenious Studies
Sabinene	0.41	0.2 [52]
Alpha-Pinene	1.66	2.67 [64]
I-beta-Pinene	0.49	0.3 [52]
β-Pinene	0.24	0.71 [65]
α-Terpinene	9.09	7.69 [64]
Eucalyptol	5.03	-
γ-Terpinene	22.66	19.54 [64]
Terpinolene	1.76	3.09 [64]
Terpinen-4-ol	53.98	40.44 [64]
α-Gurjunene	0.30	0.2 [52]
(+)-Gurjunene	0.86	-
Aromadendrene	0.31	1.5 [52]
δ-Cardinene	0.28	1.3 [52]
β -Gurjunene	1.73	0.1 [52]
δ-Cardinene	1.22%	1.3 [52]

**Table 2:** Compounds extracted from *M. bracteata* in different studies.

Identified Compounds	Quantity (%) [62]	Quantity (%) [69]
alpha-phellendrene	0.49%	0.1%
p-Mentha-2,8-diene-1-ol	0.92%	-
Terpinolene	0.64%	0.3%
Linalool	0.50%	1.1%
Methyl cinnamate	0.77%	11.4%
Methyl eugenol	96.02%	82%
Germacrene D	0.67%	0.2%

## Data Availability

Not applicable.
